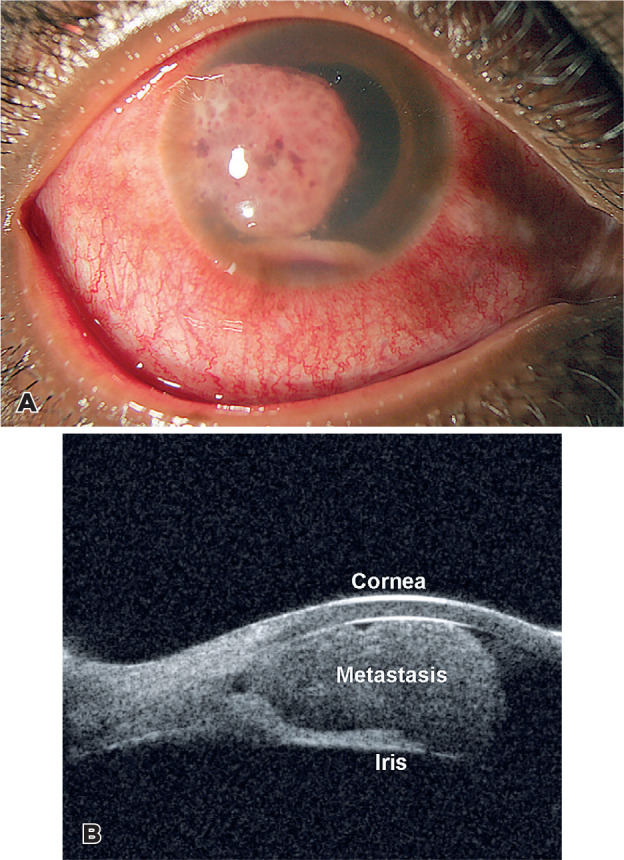# Anterior chamber metastasis from adenocarcinoma of the uterine cervix

**DOI:** 10.5935/0004-2749.2024-0358

**Published:** 2025-02-11

**Authors:** J. William Harbour, Zélia Maria Corrêa

**Affiliations:** 1 Ocular Oncology Service, Department of Ophthalmology, and Simmons Comprehensive Cancer Center, University of Texas Southwestern, Dallas, TX, USA; 2 Ocular Oncology Service, Bascom Palmer Eye Institute and Sylvester Comprehensive Cancer Center, University of Miami, Miller School of Medicine, Miami, FL, USA

A 32-year-old woman with multiorgan metastatic cervix adenocarcinoma reported pain and blurred vision in the right eye (OD). Her vision was counts fingers with an intraocular pressure of 30 mmHg OD. Figure 1A depicts the fleshy vascularized iris mass with layered hyphema and pseudohypopyon. The iris mass measuring 6.9 × 6.8 × 3.3 mm on 50 MHz ultrasonography is shown in Figure 1B. Choroid OD and left eye were normal. She expired prematurely despite systemic chemotherapy. Cervix cancer is the second most prevalent malignancy in women rarely metastasizing to the iris^(1^,^2)^.

**Figure:**